# Doyne honeycomb retinal dystrophy – functional improvement following subthreshold nanopulse laser treatment: a case report

**DOI:** 10.1186/s13256-018-1935-1

**Published:** 2019-01-10

**Authors:** Andrea Cusumano, Benedetto Falsini, Emiliano Giardina, Raffaella Cascella, Jacopo Sebastiani, John Marshall

**Affiliations:** 1 University of Rome Tor Vergata, Via Montpellier 100133, Rome, Italy; 2 Department of Ophthalmology, Catholic University of Rome, Rome, Italy; 3 Molecular Genetics Laboratory UILDM, Santa Lucia Foundation, Rome, Italy; 4 Department of Ophthalmology, Tor Vergata University of Rome, Rome, Italy; 5 UCL Institute of Ophthalmology, University College, London, UK

**Keywords:** Doyne honeycomb retinal dystrophy (DHRD), Age-related macular degeneration, Subthreshold nanosecond laser treatment

## Abstract

**Background:**

Based on phenotypic similarities between age-related macular degeneration and the autosomal disorder Doyne honeycomb retinal dystrophy, we report on a single nanolaser treatment of a patient with genotype Doyne honeycomb retinal dystrophy confirmation and evidence of disease progression over 12 months. The case study is the first report of short-term results of subthreshold nanolaser treatment in a patient with Doyne honeycomb retinal dystrophy.

**Case presentation:**

A 43-year-old Caucasian man with moderate loss of visual acuity in his left eye (20/40) and normal visual acuity in his right eye (20/20), with clinical Doyne honeycomb retinal dystrophy diagnosis and genetic confirmation of the common heterozygous mutation (*EFEMP1*) by genetic testing, underwent nanopulse subthreshold laser treatment in his left eye.

A safety examination, carried out 7 days after treatment, and clinical follow-up, conducted 60 days following laser treatment, showed improvement of visual acuity from baseline by two letters and a subjective improvement of blurring. While no apparent morphological changes were found on fundoscopy, increased autofluorescence in the treated eye was observed on imaging. In addition, 2 months after nanopulse subthreshold laser treatment, rod-mediated and cone-mediated full-field electroretinography b-wave amplitudes showed an increase from baseline in both the treated eye (300%) and untreated eye (50%). At 2 months after nanopulse subthreshold laser treatment, multifocal electroretinograms showed improvement. Acuity and full-field electroretinography improvement persisted at 6-month follow-up.

**Conclusions:**

Sustained improvements in retinal function on electroretinography persisted in both eyes 6 months after treatment, suggesting an enhancement of phototransduction and retinoid recycling induced by nanopulse subthreshold laser treatment. The functional improvement observed in the untreated eye is hypothesized to arise from an increased expression and release of metalloproteinases that circulate systemically.

## Background

Doyne honeycomb retinal dystrophy (DHRD), also known as Malattia Leventinese, Online Mendelian Inheritance in Man (OMIM) 126600, is an autosomal dominant disorder caused by a single missense mutation, Arg345Trp (R345W), in the gene EGF containing fibulin-like extracellular matrix protein 1 (*EFEMP1*) [1–4].The disease is typically characterized by early-onset drusenoid deposits involving the posterior pole and the peripapillary area, often with a radial distribution. It has been suggested that mutant *EFEMP1* may alter the extracellular matrix in Bruch’s membrane, leading to the accumulation of basal laminar deposits [1, 3]. Evidence for this lies in *in vivo* retinal microanatomy imaging by time-domain optical coherence tomography (OCT) showing diffuse alterations of the retinal pigment epithelium (RPE) and Bruch’s membrane, with preservation of the neurosensory layers [5–7]. In addition, mutations in the gene encoding tissue-inhibitor metalloproteinase-3 (*TIMP3*) are tightly linked to *EFEMP1* and are associated with a severe form of DHRD [8–10]. Likewise, age-related macular degeneration (AMD) in its intermediate stage is characterized by drusen or drusenoid deposits, Bruch’s membrane thickening, and RPE atrophy (see review, Hulleman [11]). AMD and DHRD also share pathophysiologic similarities: two mouse models of DHRD showed complement activation and RPE atrophy, akin to the pathology observed in AMD [11, 12].

In 2013, Lenassi *et al.* showed that low-energy argon laser treatment performed just outside the drusen boundaries, but away from the fovea, induced drusen clearance and improved visual function in patients with DHRD with confirmed *EFEMP1* mutation [13]. Recently, a low-energy, subthreshold nanosecond laser, the 2RT® (Ellex, Adelaide, Australia), has been utilized to induce targeted and controlled RPE injury without significant retinal neuronal damage or gliosis [14–16]. A clinical trial in intermediate AMD provided evidence of efficacy at 12-month follow-up, both in the treated and untreated eye, with improvement in flicker sensitivity on electroretinography (ERG) and reduction in the mean drusen area [17]. A similar effect was found in the APoE-null mouse model of AMD [18], with thinning of Bruch’s membrane and increased expression of matrix metalloproteinase-2 and matrix metalloproteinase-3 following nanolaser treatments.

Based on the phenotypic similarities between AMD and DHRD, a single application of nanopulse subthreshold laser treatment (NSLT) was attempted in a patient with Arg345Trp (R345W) genotype confirmation and clear evidence of disease progression over the previous 12 months. Treatment was delivered in one eye (the worse eye) with a modification to the standardized protocol according to Guymer *et al.* [17], applying 24 nanosecond laser spots. Retinal structure and function were assessed before and after the treatment. Results showed no appreciable changes in retinal morphology but an increase in autofluorescence on imaging in the treated eye. There was also a functional improvement in both the treated and untreated eye on ERG. This is the first report following NSLT of DHRD.

## Case presentation

**Fig. 1:**
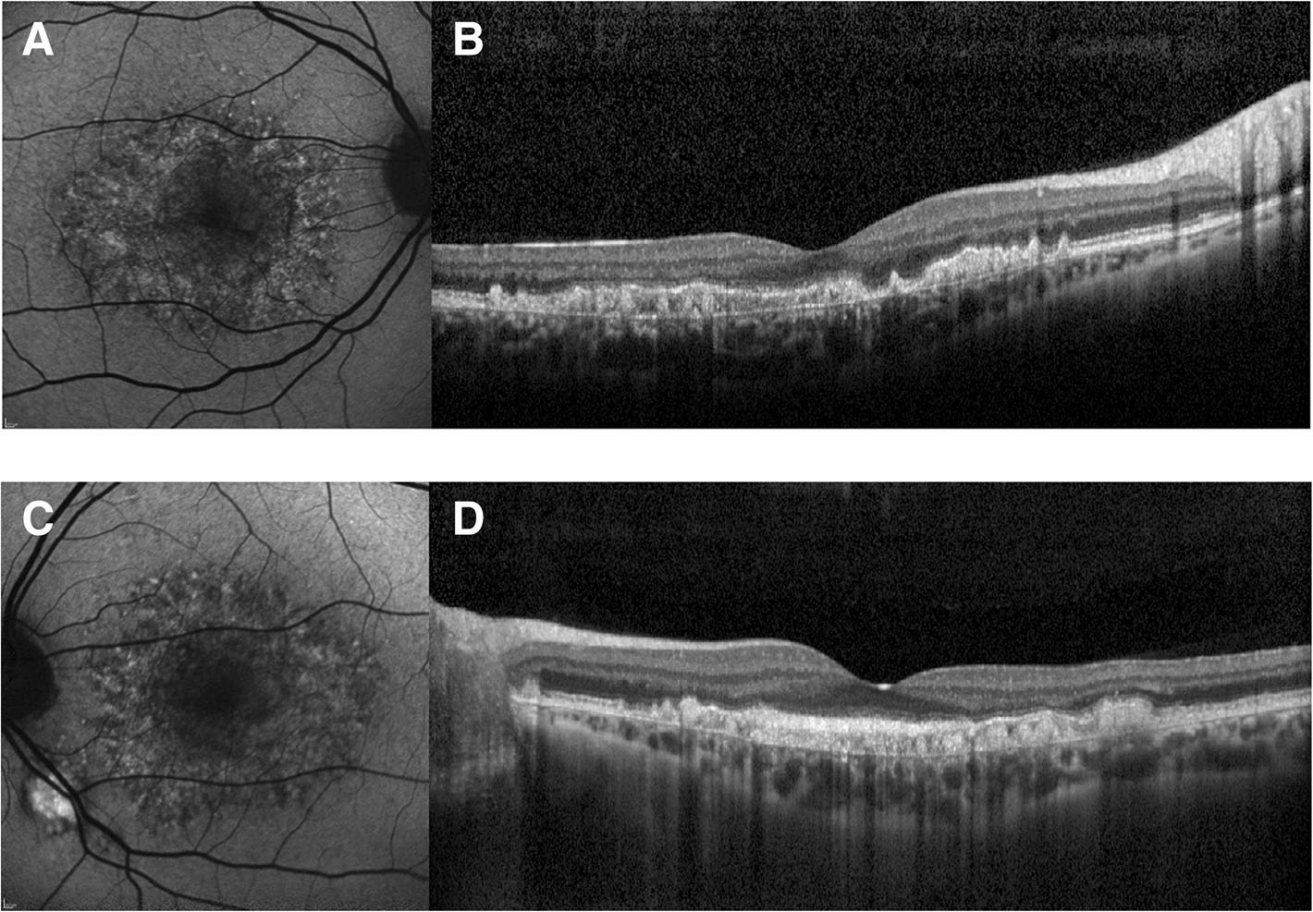
**a**, **b** Right eye, autofluorescence and optical coherence tomography imaging before 2RT® laser treatment. **c**, **d** Left eye, autofluorescence and optical coherence tomography imaging after 2RT® laser treatment

A 43-year-old Caucasian man presented with a moderate loss of visual acuity in his left eye (20/40) and normal right eye acuity (20/20). His medical, family, and psychosocial history was irrelevant. He did not have a history of medication use or previous diseases other than common childhood infectious diseases. For 12 months he complained of blurring, progressive alteration of night vision, and reduced contrast sensitivity in both eyes, with a much more pronounced effect in his left eye. Clinical diagnosis of DHRD was made after full ophthalmologic examination and detailed retinal imaging. Figure 1 shows OCT and fundus autofluorescence in both eyes. ERG, including mesopic and photopic full-field ERGs as well as multifocal ERGs (mfERG), were performed at baseline and 7 days after treatment. Genetic analysis confirmed the common heterozygous DHRD mutation in *EFEMP1*: (2p16.1) (p.R345W; c.1033C > T).

**Fig. 2:**
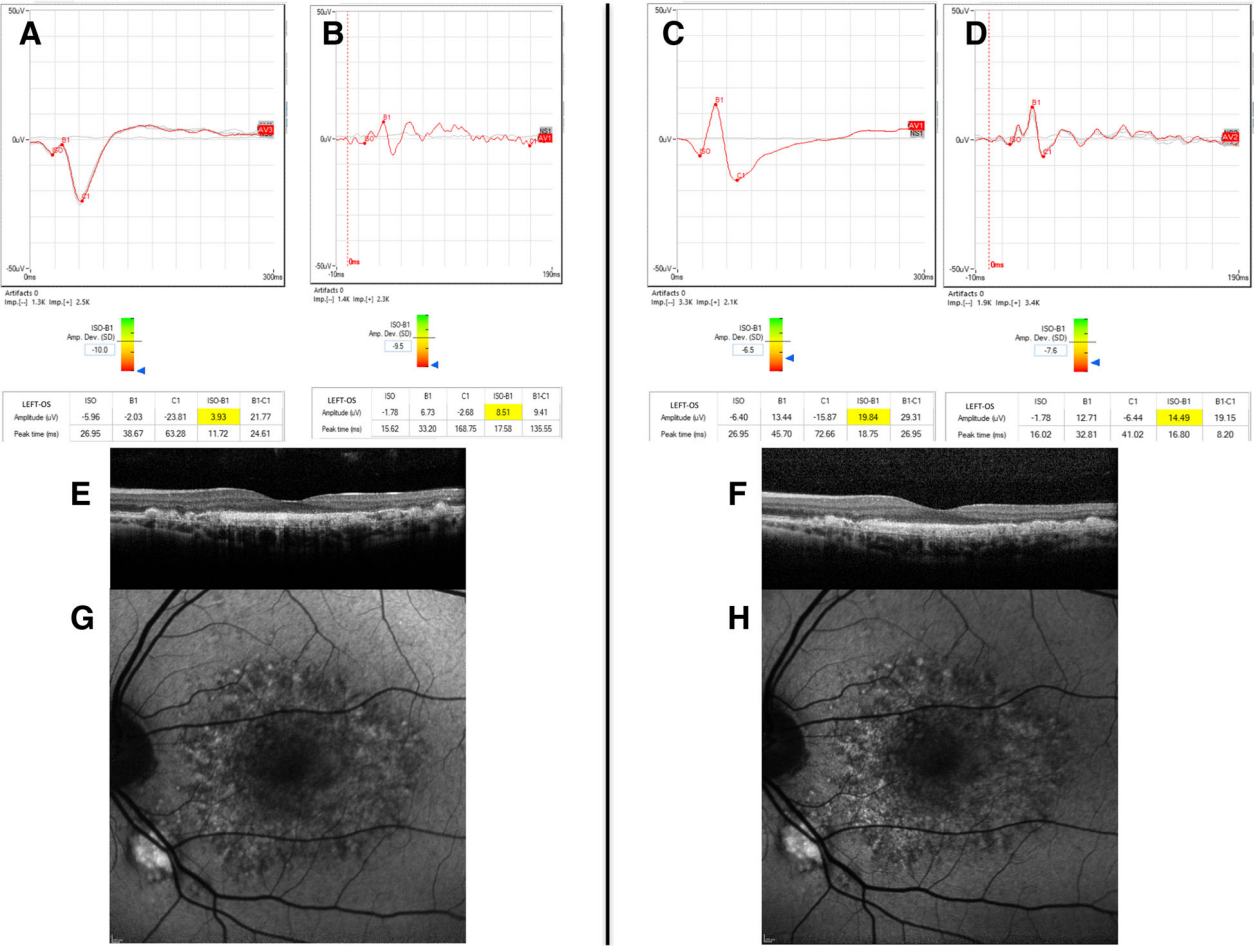
**a, b** Mesopic and photopic electroretinography b-wave responses before 2RT® laser treatment. **c**, **d** Mesopic and photopic electroretinography b-wave responses 6 months after 2RT® laser treatment. Notice the large increase in the b-wave amplitude. **e**, **f** Optical coherence tomography imaging of the patient before and 1 month after 2RT®laser treatment, respectively. No significant changes in microanatomy were observed. **g**, **h** Autofluorescence imaging of the patient before and 1 month after 2RT®, respectively. A moderate increase in retinal autofluorescence was observed

**Fig. 3:**
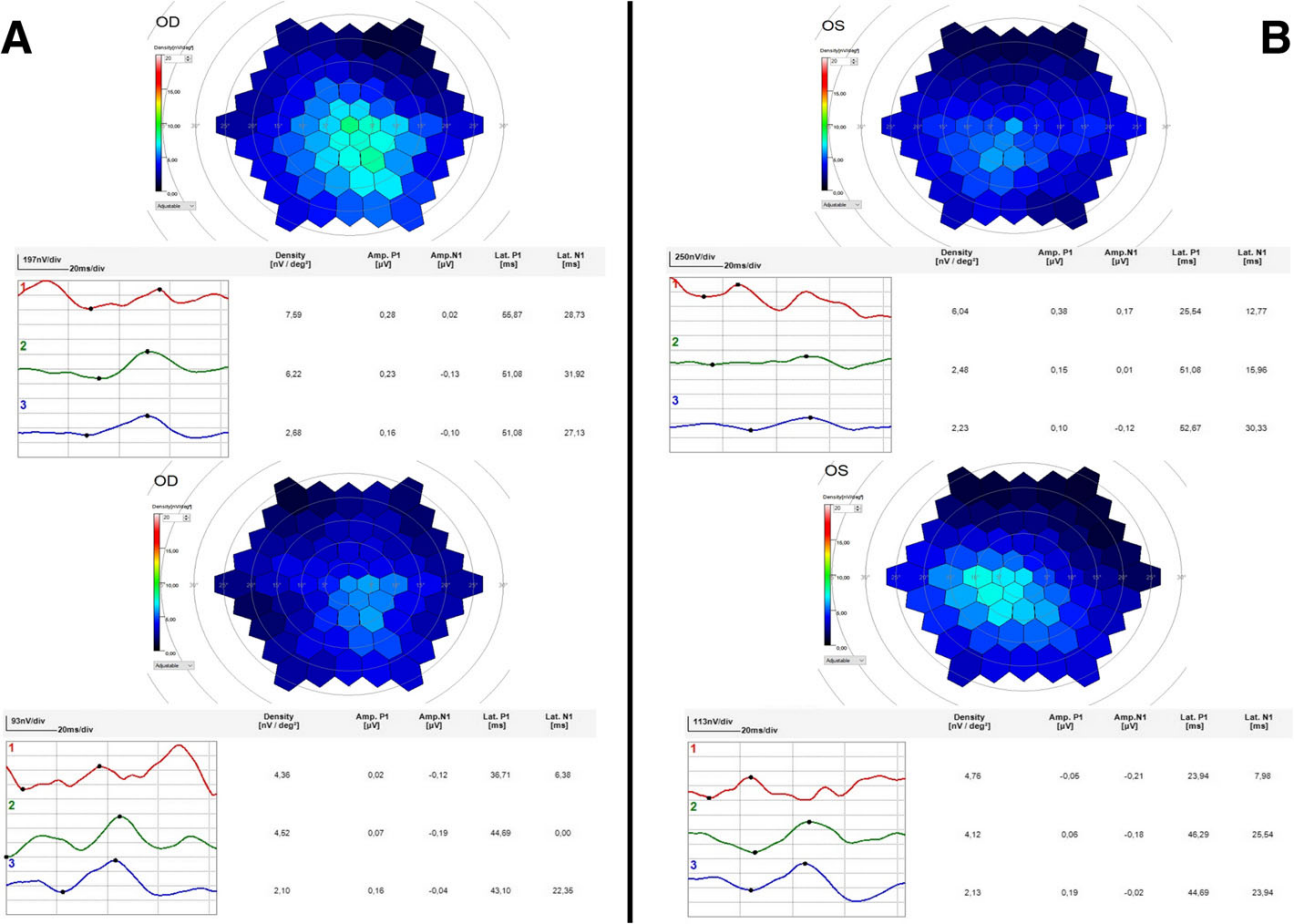
**a** Multifocal electroretinography in the right eye (fellow-eye). On top: amplitude and implicit time before 2RT® laser treatment in the left eye (contralateral eye treated). Bottom: 2 months after treatment in the left eye (contralateral eye treated). **b** Multifocal electroretinography in left eye (treated eye). On top: amplitude and implicit time before 2RT® laser treatment in the left eye (treated eye). Bottom: 2 months after treatment in the left eye (treated eye). OD right eye, OS left eye

He was offered NSLT for his left eye. After written informed consent and full explanation of methods and procedure were completed, he underwent the treatment in his left eye, which applies ultra-low energy laser pulses in 24 spots around the macula of one eye (0.15–0.45 mJ), using 400 μm diameter laser spots, 3 nanosecond pulse length, 532 nm wavelength and energy titrated to the patient. Clinical follow-up was conducted at 60 and 180 days after treatment. Table 1 summarizes the clinical ophthalmological testing performed during follow-up. Visual acuity improved from baseline by two letters. There was a subjective improvement in blurring in his left eye. No morphological changes were apparent on fundoscopy, but increased autofluorescence in the treated eye was observed on imaging (Fig. 2). Rod-mediated and cone-mediated ERG b-wave amplitudes showed an increase from baseline in both the treated eye (300%) and fellow eye (50%) (Fig. 2). mfERG amplitudes did not change significantly from baseline, but the implicit time of the main positive component decreased by 8 milliseconds compared to baseline in the treated eye and by 5 milliseconds in the fellow eye (Fig. 3). Subjective and clinical improvements persisted unchanged at 6-month follow-up. The rod-mediated and cone-mediated ERG b-wave amplitude remained unchanged (300% increase) in the treated eye and returned to the pre-treatment value in the fellow eye.

**Table 1:** Summary of the clinical ophthalmological testing performed during follow-up

OD	Baseline	2 Months	6 Months
Visual acuity	84	85	85
OCT (CRT)	266	275	282
Autofluorescence	No change	No change	No change
Mesopic	8.59	5.36	10.26
Photopic	10.06	11.67	11.36
PHNR	5.72	6.18	6.08
OS	Baseline	2 Months	6 Months
Visual acuity	59	61	61
OCT (CRT)	323	331	274
Autofluorescence	No change	No change	No change
Mesopic	3.93	11.11	19.84
Photopic	8.51	11.54	14.49
PHNR	6.21	8.8	6.06

*CRT* central retinal thickness *OCT* optical coherence tomography, *OD* right eye, *OS* left eye, PHNR photopic negative response

## Discussion and conclusion

To the best of our knowledge, this is the first report describing the short-term results of NSLT in DHRD. The treatment was well tolerated and transpired without adverse events or complications up to the 6-month post-intervention time point, with sustained improvement in retinal function on ERG in both eyes. The improvement in ERG testing suggests enhanced phototransduction and retinoid recycling and aligns with the mechanism of action of this treatment, which in preclinical models was shown to reduce the thickness of Bruch’s membrane and increase the expression of metalloproteinases, thus resulting in improved retinoid recycling. The functional improvement observed in the untreated eye is consistent with other studies on this device and is hypothesized to arise from an increased expression and release of metalloproteinases that circulate systemically. The present results encourage further long-term studies with the subthreshold nanosecond laser as a potential treatment of retinal and RPE abnormalities associated with DHRD and similar conditions such as AMD.
